# Extracutaneous Sporotrichosis With Dissemination to the Brain in a Patient Taking Biologic Medications: A Case Report

**DOI:** 10.7759/cureus.95847

**Published:** 2025-10-31

**Authors:** Kailey N Nguyen, Donna Defreitas, Bryan Youree, Shovendra Gautam

**Affiliations:** 1 Internal Medicine, Baylor Scott & White All Saints Medical Center, Fort Worth, USA; 2 Infectious Disease, Baylor Scott & White All Saints Medical Center, Fort Worth, USA

**Keywords:** chronic use of biologics, disseminated extracutaneous sporotrichosis, disseminated sporotrichosis, immunosuppressed patient, interleukin-23 inhibitor, rheumatoid arthritis, sporothrix schenckii fungus

## Abstract

Sporotrichosis is a fungal infection caused by *Sporothrix *species, primarily *Sporothrix schenkii *and *Sporothrix brasiliensis. *Disease manifestation varies from localized cutaneous form to disseminated cutaneous and disseminated extra-cutaneous forms. We present a case of sporotrichosis with widespread skin lesions and fungal dissemination to the brain in a patient taking biologic medications to treat psoriatic arthritis. Her diagnosis was made via positive skin tissue culture, and her symptoms responded well to amphotericin B. With this case report, we would like to highlight the risk of rare and severe infection, such as sporotrichosis with dissemination to the central nervous system, in patients with immunosuppression secondary to biologic medications.

## Introduction

Sporotrichosis is a fungal infection caused by inoculation of species of dimorphic *Sporothrix*, among which the most virulent species are *Sporothrix schenkii *and *Sporothrix brasiliensis* [1]. *Sporothrix schenkii* lives in warm and humid environments and is classically associated with rose bushes and gardeners. However, they have also been isolated from different flowers, maize roots, and leaves since they live in soil and an environment high in cellulose, such as grasses, wood, leaves, and branches [1]. The primary route of entry is through traumatic cutaneous injury, followed by an incubation period of three weeks [1]. Less commonly, the respiratory route is also reported [2].

Risankizumab is an interleukin-23 inhibitor that is indicated for moderate to severe plaque psoriasis [3]. Adalimumab is a tumor necrosis factor alpha blocker also used for moderate to severe chronic plaque psoriasis [4]. Due to the immunosuppressant property of these biologic medications, they are known to increase the risk of infection [3-4].

Here we report a case of sporotrichosis in a patient with a history of psoriatic arthritis who was taking adalimumab when she contracted the mycotic disease. Our patient was diagnosed with extracutaneous sporotrichosis involving the central nervous system, which is estimated to affect 0.1% of all *Sporothrix *infections [5]. Even though she was on a biologic medication that potentially could increase her risk of severe infection, her presentation is also unique in that she did not develop any neurological symptoms. In her case, the dissemination to the brain was diagnosed 2.5 months from the onset of cutaneous symptoms.

## Case presentation

**Figure 1:**
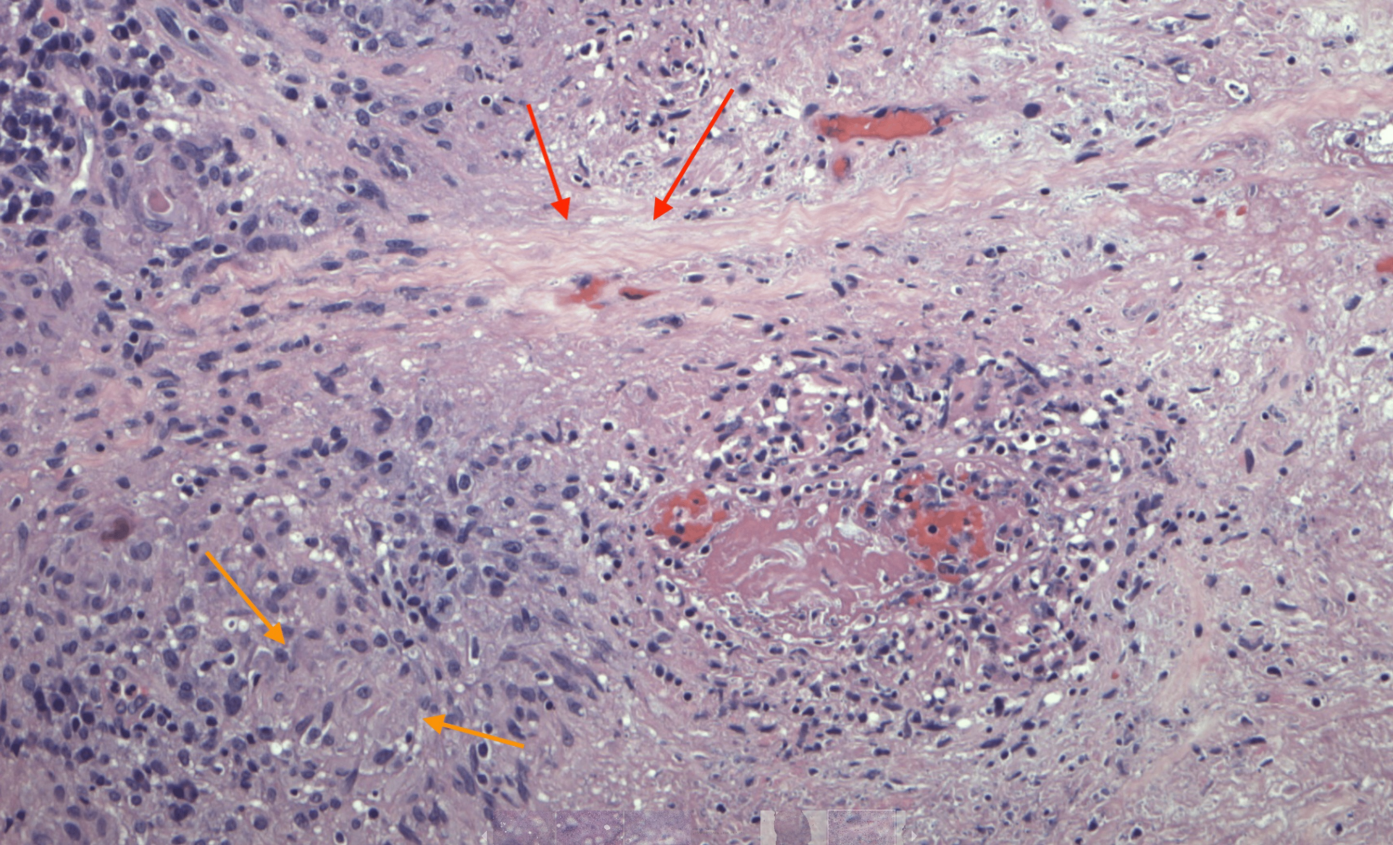
Microscopy examination of the lesion. Granulomatous inflammatory infiltrate (orange arrows) with surrounding neutrophils and focal necrosis (red arrows).

Our patient is a 61-year-old female with a medical history significant for psoriatic arthritis, asthma, hypothyroidism, and hyperlipidemia. She recalled that as early as March of 2025, she noticed right arm swelling and pain. Thinking it was related to her psoriatic arthritis, she started wearing a splint to reduce the swelling. She also developed cutaneous knots along her tricep. Around the same time, she had a rash on her forehead, which was biopsied by her dermatologist. Histological result of the biopsies returned significant for granulomatous dermatitis with neutrophil infiltrates and focal necrosis, highly suspicious for an infectious process (Figure 1). The granulomatous infiltrate also extended into the subcutaneous tissue.

When the rash first appeared, the patient was three doses into a weekly tumor necrosis factor alpha blocker, adalimumab, regimen to treat her psoriatic arthritis. Prior to adalimumab, the patient was managed on the interleukin 23 inhibitor risankizumab since the diagnosis in 2024. Other home medications included montelukast for asthma, and vitamin D and vitamin C supplements. She lived on a farm, mostly interacted with goats, handled the hay, and reported occasional skin scratches from those daily activities. The patient also reported having rose bushes on her property, but denied frequent interaction with them.

**Figure 2:**
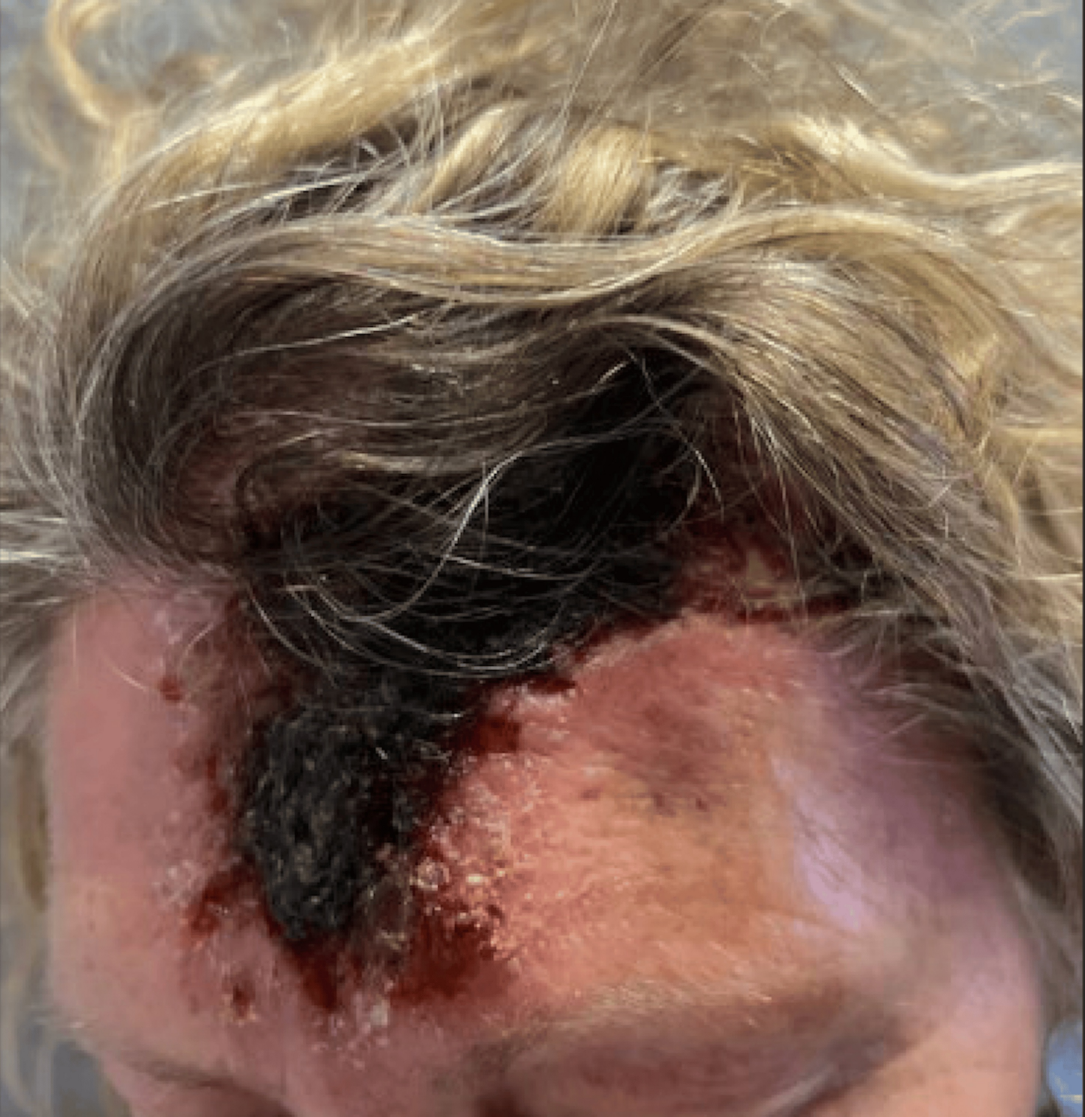
Forehead lesion. The lesion reportedly grew larger after biopsy.

**Figure 3:**
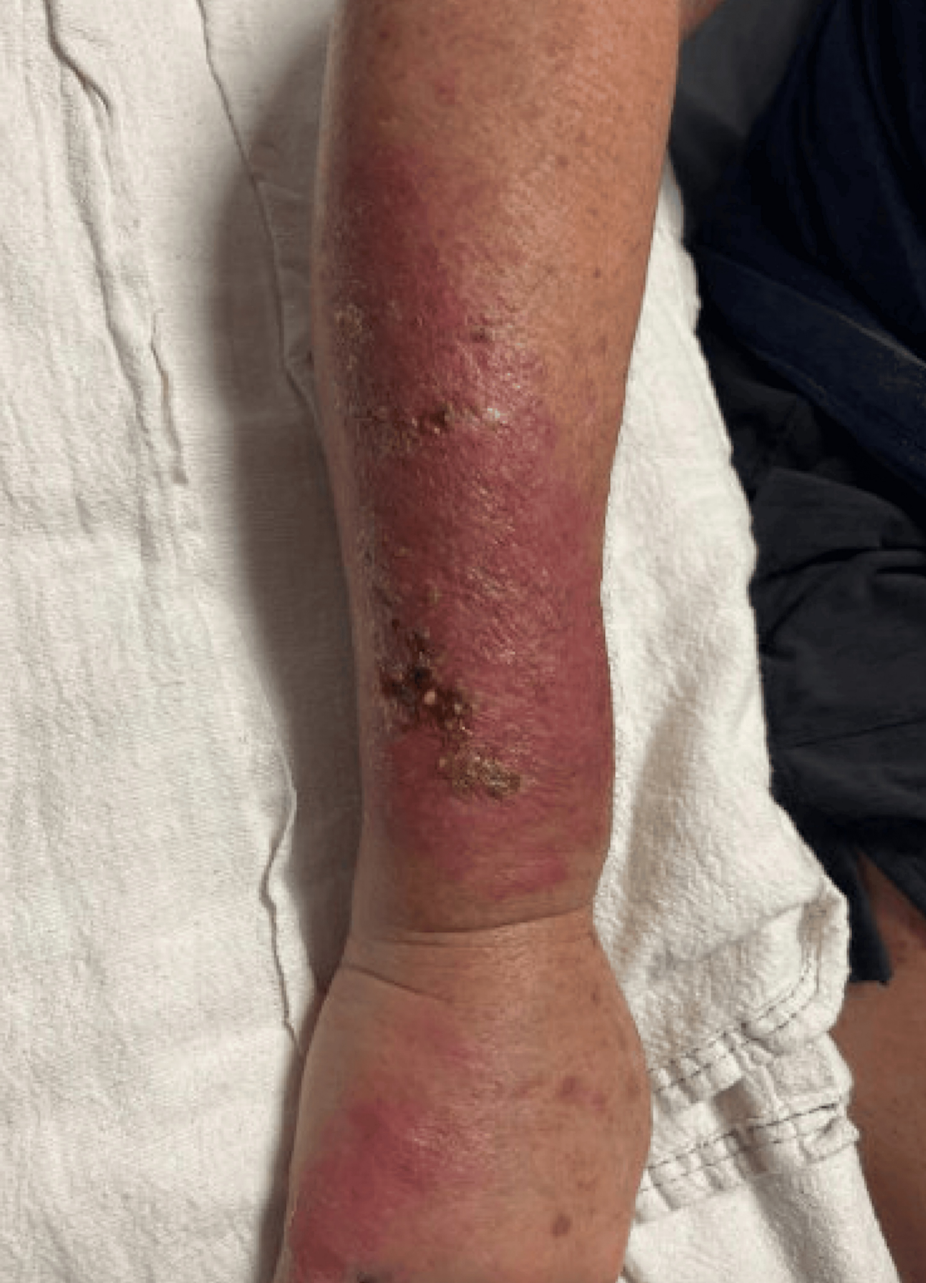
Right arm lesion. Initial area of swelling slowly became erythematous, then turned into area of dry, scaly rash.

The rash on her forehead reportedly grew larger in size after the biopsy (Figure 2). The swollen area on her right arm slowly became erythematous and turned into a dry, scaly rash (Figure 3), which prompted a hospital admission where she was treated with a course of intravenous vancomycin for suspected cellulitis. The rash improved, and the patient was discharged without further antibiotics.

Soon after the initial hospital discharge, her right upper arm rash returned, with swelling, erythema, ulceration, and pus drainage. This prompted an outpatient course of antibiotics with sulfamethoxazole-trimethoprim as well as a short course of daily prednisone 20 mg. When her symptoms did not resolve, the patient was referred back to the hospital. During this hospitalization, which was 4 weeks after the initial appearance of the right arm swelling, she underwent a repeat biopsy of the skin lesion. The rash was not believed to be of infectious etiology, and she was discharged with a short course of oxycodone for pain and instructed to follow up with her dermatologist while the biopsy results were pending. Culture results of the biopsies returned positive for *Sporothrix schenckii*. The patient was treated in the outpatient setting with 4 weeks of itraconazole for sporotrichosis. The lesions stabilized but did not improve after the course of itraconazole. At this point, which was about 2.5 months after the initial appearance of the swelling on the right arm, she was admitted to the hospital for disseminated sporotrichosis.

Upon this hospitalization’s initial presentation, vital signs (Table 1) and blood test results (Table 2) were mostly within normal limits.

**Table 1:** Vital signs.

Vitals	Patient	Normal Range
Blood pressure	114/71	Less than 120/80 mmHg
Pulse rate	66	60 to 100 beats per minute
Respiratory rate	16	12 to 20 breaths per minute
Arterial Oxygen Saturation	97% on room air	95 - 100 %

**Table 2:** Blood test results

Blood Test	Patient's Value	Normal Range
Comprehensive Metabolic Panel		
Glucose	107 (H)	70 - 99 mg/dL
Sodium	135 (L)	136 - 145 meq/L
Potassium	4.1	3.6 - 5.0 meq/L
Chloride	102	98 - 107 meq/L
Carbon Dioxide	33 (H)	21 - 32 meq/L
Blood urea nitrogen (BUN)	12	7 - 18 mg/dL
Creatinine	0.91	0.55 - 1.02 mg/dL
BUN/Creatinine Ratio	13	7 - 25
Estimated Glomerular Filtration Rate (eGFR)	72	>=60 mL/min/1.73m2
Calcium	9.9	8.5 - 10.1 mg/dL
Total Protein	7.8	6.4 - 8.2 g/dL
Albumin	3.4	3.4 - 5.0 g/dL
Globulin (Calculated)	4.4 (H)	1.7 - 3.3 g/dL
Complete Blood Count		
White Blood Cell Count	5.5	4.5 - 11.0 10*3/uL
Red Blood Cell Count	4.11	4.00 - 5.40 10*6/uL
Hemoglobin	12.6	12.0 - 16.0 g/dL
Hematocrit	39.0	37.0 - 47.0 %
Mean corpuscular volume (MCV)	94.9	80.0 - 99.0 fL
Platelet Count	341	150 - 450 10*3/uL
Absolute Neutrophil Count (ANC)	4.18	2.07 - 8.80 10*3/uL
Lymphocytes, Absolute	0.83	0.72 - 4.32 10*3/uL
Monocytes, Absolute	0.41	0.09 - 0.99 10*3/uL
Eosinophils, Absolute	0.02	0.00 - 0.76 10*3/uL
Basophils, Absolute	0.05	0.00 - 0.22 10*3/uL

**Figure 4:**
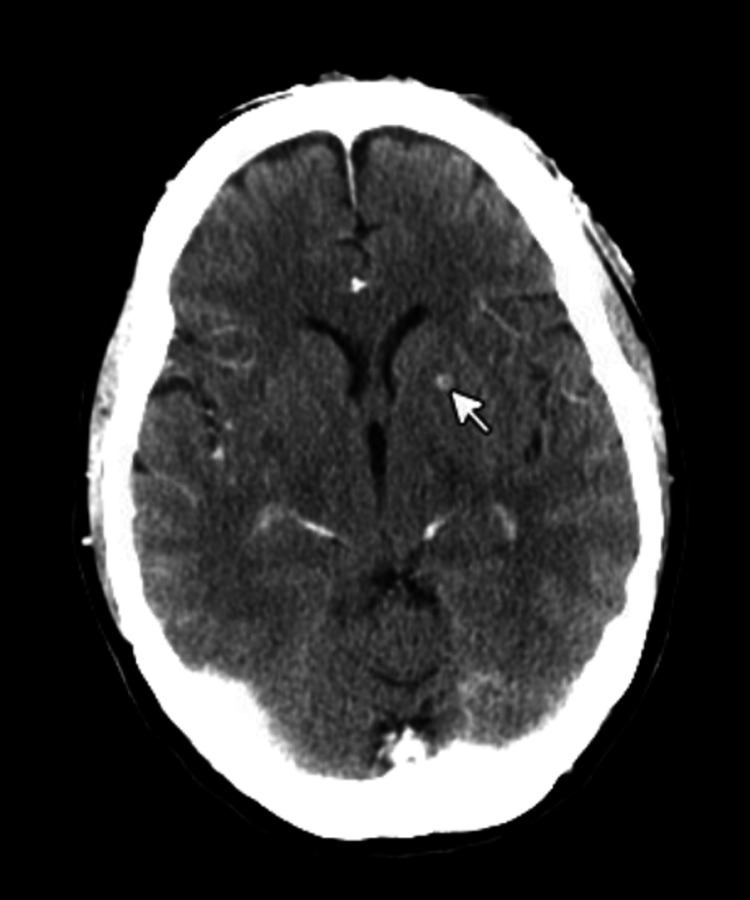
CT of the head. Area of abnormal parenchymal enhancement with central low attenuation within the left lentiform nuclei (white arrow).

**Figure 5:**
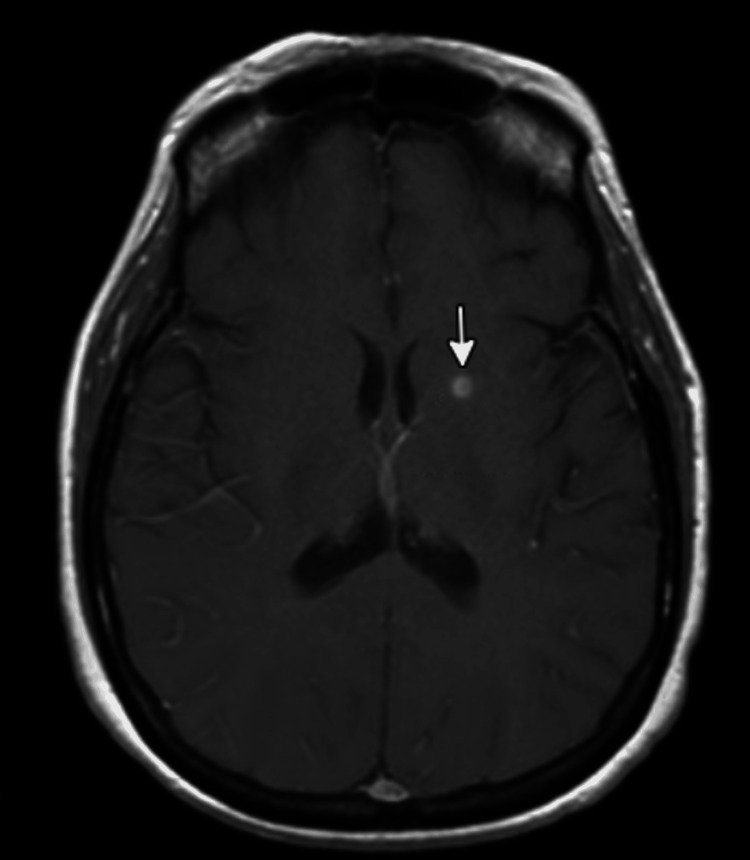
T1-weighted MRI with and without gadolinium contrast showing lesion concerning for infectious etiology. Ring-enhancing lesion within the left lentiform nuclei (white arrow).

She never developed any neurological symptoms; however, due to the concern of cerebral extension from the cutaneous rash on the forehead, imaging of the head was done upon presentation to the emergency department. Computed tomography (CT) of the head shows a 3.5 mm area of abnormal parenchymal enhancement with central low attenuation within the left lentiform nucleus (Figure 4). Follow-up magnetic resonance imaging (MRI) of the brain was performed to further characterize the lesion. T1-weighted MRI image with gadolinium contrast showed ring-enhancing lesions within the left lentiform nuclei and right occipital lobe, as well as small leptomeningeal versus cortical foci of enhancement within the right parietal lobe, favoring an infectious etiology (Figure 5).

**Figure 6:**
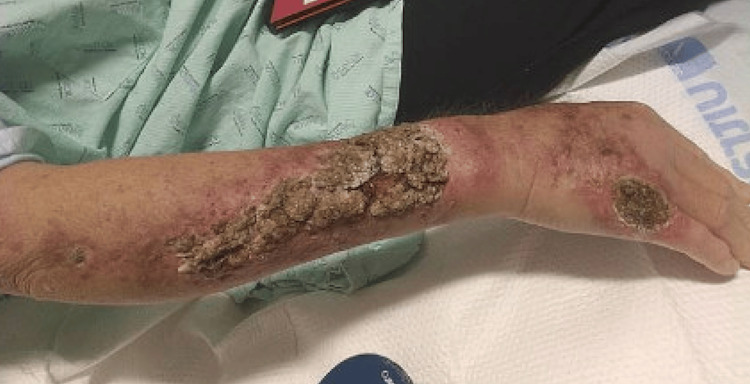
Right arm lesion before treatment with amphotericin B.

**Figure 7:**
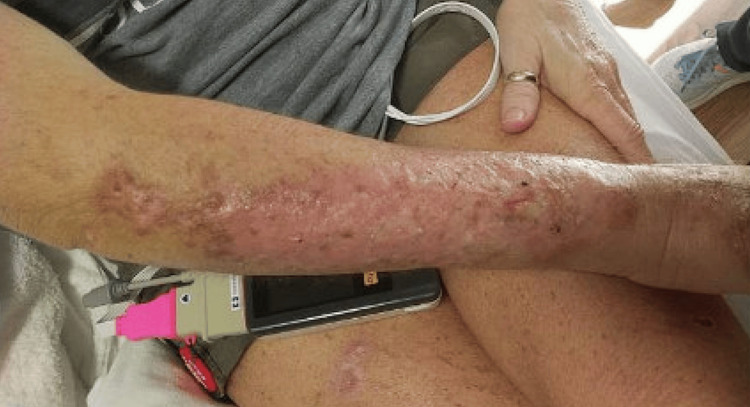
Right arm lesion after treatment with amphotericin B.

**Figure 8:**
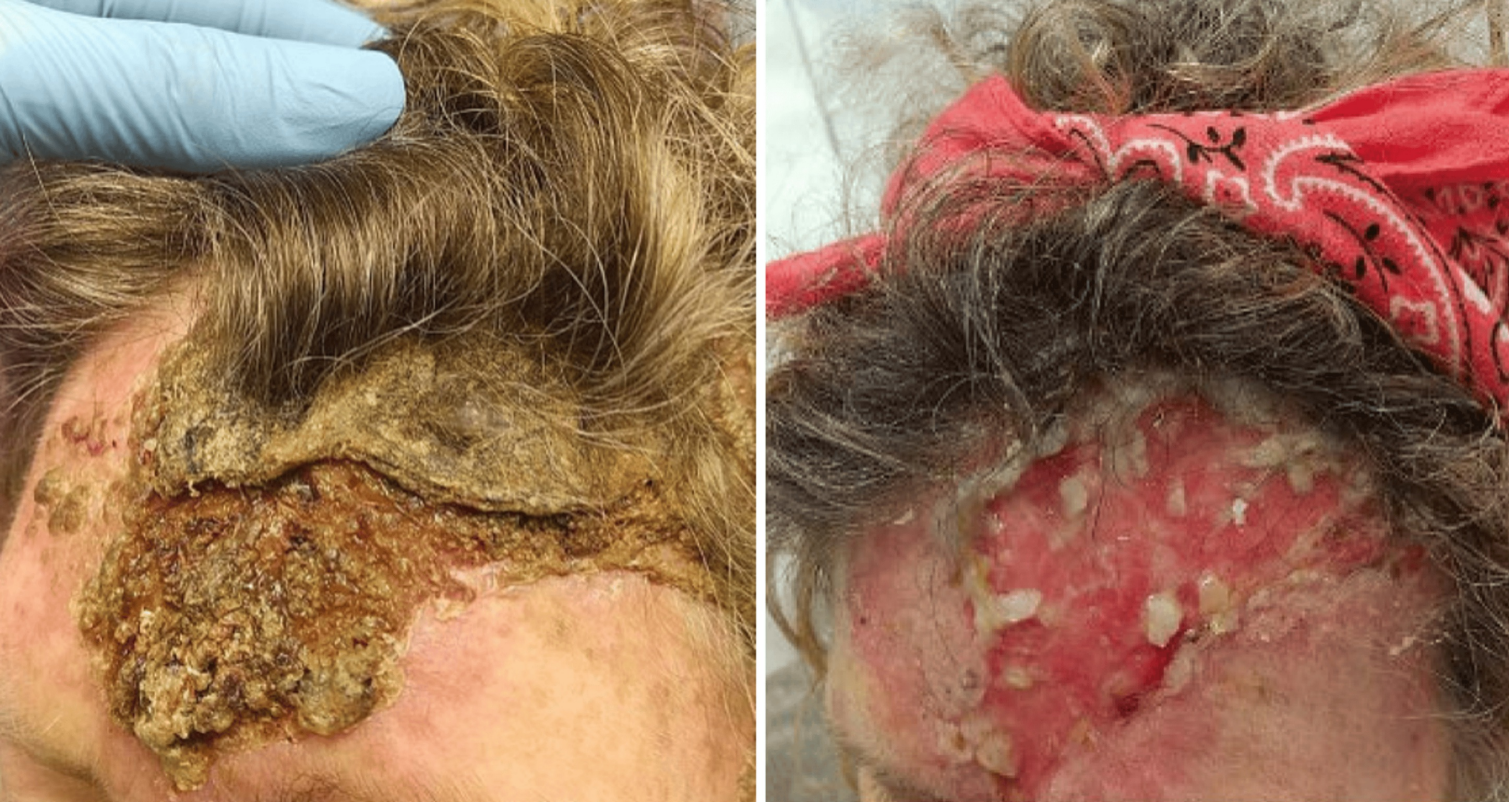
Comparison of forehead lesion before (left) and after (right) treatment with amphotericin B.

The patient was diagnosed with extra-cutaneous disseminated sporotrichosis and placed on amphotericin B for 14 days. Her dermal lesions improved (Figures 6, 7, 8). Repeat MRI of the brain 7 days after showed no significant changes. Upon discharge, the patient was placed on a regimen of oral posaconazole due to concern that she failed itraconazole therapy. She was to remain on the anti-fungal maintenance therapy for 12 months.

## Discussion

Sporotrichosis is a mycotic infection caused by *Sporothrix* species, commonly *Sporothrix schenckii* [6]. It is considered an occupational disease among farmers and gardeners in endemic areas such as Mexico, Central America, and Brazil [6]. Sporotrichosis manifestations are mainly limited to the skin. The cutaneous form is described as one or a few lesions clustered at the inoculation site [2].

Disseminated sporotrichosis, a severe and rare form of disease manifestation, often occurs in immunosuppressed patients and is estimated to account for 1.3% of all cases of sporotrichosis [7]. Disseminated cutaneous sporotrichosis involves multiple lesions in noncontiguous regions of the skin, whereas the disseminated extracutaneous form involves other organ systems such as lungs, bones, or meninges [1]. It is estimated that dissemination to the central nervous system (CNS) occurs in 0.1% of all cases of *Sporothrix *infections [5]. Patients with CNS involvement commonly present with symptoms like headache, nuchal rigidity, cognitive changes, seizures, cranial nerve palsies, and motor deficits [8]. Common imaging findings include meningeal contrast enhancement, hydrocephalus, hemorrhagic or ischemic lesions [8]. The rate of mortality for sporotrichosis involving the central nervous system is estimated to be as high as 57% [9].

Interleukin-23 inhibitors and TNF-alpha inhibitors are classes of biologic medications used to manage psoriatic arthritis. Interleukin 23 plays a role in promoting proliferation and differentiation of immune cells involved in the inflammatory response [10]. TNF alpha is another cytokine involved in the inflammatory response, playing a role in cell proliferation and differentiation, recruiting immune cells, and initiating the inflammatory cascade [11]. By inhibiting key processes in the immune system, these medications downregulate processes contributing to autoimmune diseases. However, via the same process, they also suppress the autoimmune system and increase the risk of infections.

Disseminated sporotrichosis is often associated with chronic alcoholism or an immunosuppressed state, such as infection with human immunodeficiency virus. Our patient tested negative for HIV. Although she denies frequent alcohol use, being on a biologic regimen for psoriasis, she presents with a major risk factor for severe infection. Initially, she was treated for cellulitis out of concern for her immunosuppressed state. However, after being on multiple antibiotics without improvement, the tissue culture was finally obtained and returned positive for *Sporothrix schenckii.* After failing the initial course of oral antifungal medication, which was thought secondary to the overwhelming infection in the setting of an immunosuppressed state, the patient was put on a 14-day course of amphotericin B, which produced a good clinical response. Additionally, evidence of disease dissemination to her brain was found in less than 2.5 months after the first sign of cutaneous disease manifestation. This emphasizes the importance of keeping sporotrichosis high on the differential for immunosuppressed patients presenting with ulcerative cutaneous lesions, especially in endemic areas and in those with occupational risk factors such as farmers.

## Conclusions

Disseminated sporotrichosis is a severe mycotic infection that is primarily associated with chronic alcoholism and an immunosuppressed state, such as HIV infection. Here we present a case of extra-cutaneous sporotrichosis with dissemination to the central nervous system. Even though our patient did not have the usual risk factors, such as HIV infection or alcoholism, her immune system was suppressed secondary to biologic medications for psoriatic arthritis. In her case, it took less than 2.5 months from the first sign of cutaneous symptoms to the discovery of the brain lesions. Even though she did not develop any neurological symptoms and her lesions responded well to amphotericin B, given the severity of the disease and high mortality rate, we would like to highlight the importance of keeping sporotrichosis high on the differential diagnosis for immunosuppressed patients presenting with ulcerated skin lesions, especially those refractory to antibiotics.
